# Adverse events in the neonatal intensive care unit identified by triggers

**DOI:** 10.3389/fphar.2025.1539687

**Published:** 2025-05-30

**Authors:** Fabiana Bragança Albanese, Deise de Souza Ventura, Maurício Wesley Perroud, Rafael Nogueira de Souza, Mariana Vieira Morau, Marília Berlofa Visacri, Patricia Moriel

**Affiliations:** ^1^ School of Medical Sciences, Universidade Estadual de Campinas, Campinas, Brazil; ^2^ Hospital Estadual Sumaré Dr. Leandro Franceschini, Sumaré, Brazil; ^3^ Faculty of Pharmaceutical Sciences, Universidade Estadual de Campinas, Campinas, Brazil; ^4^ Faculty of Pharmaceutical Sciences, University of São Paulo, São Paulo, Brazil

**Keywords:** adverse events, adverse drug events, trigger tool, neonatal intensive care unit, low birth weight

## Abstract

**Objective:**

The main aim of this study was to identify adverse events (AEs) in neonates admitted to a Neonatal Intensive Care Unit (NICU) using a trigger-based approach.

**Methods:**

A retrospective observational study was conducted at Hospital Estadual Sumaré -Dr. Leandro Franceschini, Sumaré, SP, Brazil, over 6 months in 2021. Data from 120 electronic medical records of neonates hospitalized for ≥48 h and prescribed at least one medication were analyzed. Seventeen triggers, such as healthcare-associated infections (HAIs), antimicrobial use, accidental extubation, electrolyte disorders, and others, were employed to identify AEs, including those specific to adverse drug reaction (ADRs). AE severity was assessed using the Neonatal Adverse Event Severity Scale (NAESS) and the World Health Organization (WHO) classification, while ADR causality was evaluated using the WHO criteria and the algorithm proposed by Du et at. Risk factors such as gestational age, birth weight, and length of hospital stay were also analyzed.

**Results:**

A total of 249 triggers identified 168 confirmed AEs, resulting in a Positive Predictive Value (PPV) of 67.5%. At least one AE was observed in 50.0% of neonates and 40.8% experienced ADRs. The most frequent triggers that identified AEs included HAIs and antimicrobial use (30.8/100 records, each), followed by hyperglycemia (22.5/100 records), increased frequency of bowel movements (16.7/100 records), and hyponatremia (10.8/100 records). Severe complications such as necrotizing enterocolitis (2.5/100 records) and accidental extubation (5.0/100 records) were also recorded. Triggers with a PPV of 100% included necrotizing enterocolitis, accidental extubation, hypocalcemia, HAIs, and antimicrobial use. According to the NAESS, most AEs were classified as grade 2 - moderate, (44.0%) or grade 3 – severe (51.2%). Critical events, such as life-threatening conditions (grade 4) and death (grade 5), were less common, totaling 4.8%. Regarding ADRs, the majority were classified as possible or unlikely by both methods. The distribution of AEs varied by neonatal subgroups, with extremely preterm showing higher rates of AEs, including hyponatremia (53.8%) and accidental extubation (66.7%). Among all events, elevated serum creatinine (75.0%), necrotizing enterocolitis (66.7%), and hypercalcemia (100.0%) predominantly occurred in neonates with extremely low birth weight (ELBW). In contrast, neonates with appropriate birth weight experienced fewer AEs and lower AE severity. This association was not assessed for gestational age.

**Conclusion:**

The findings suggest that prematurity, low birth weight, and prolonged hospitalization are relevant risk factors for AEs in NICUs. Nonetheless, trigger tools proved effective in identifying severe events and enhancing patient safety in this high-risk setting. Prevention strategies based on these findings can help mitigate risks and optimize neonatal care.

## 1 Introduction

Patient safety is an essential priority in healthcare, particularly for vulnerable populations such as neonates admitted to Neonatal Intensive Care Unit (NICU). Neonatology in compasses distinct subgroups with unique physiological characteristics influenced by ontogeny, which varies significantly across gestational ages and postnatal ages (Alcorn and McNamara, 2003). These developmental differences crucially impact pharmacokinetics, including absorption, distribution, metabolism, and excretion, thereby increasing the susceptibility of neonates to severe adverse drug events (ADEs) and drug-drug interactions (DDIs) (Tayman et al., 2011; Lim and Pettit, 2019). Additionally, the off-label and unlicensed use of medications, prevalent in NICUs (Samiee-Zafarghandy et al., 2023) due to limited availability of pediatric-specific drugs, further heightens these risks (Domingues et al., 2023). Studies have consistently reported high rates of adverse drug reactions (ADRs) in neonates, ranging from 27.4% (De Las Salas and Díaz-Agudelo, 2017) to 42.6%, with associated mortality rates reaching up to 41.6% (Kaguelidou et al., 2016). Beyond ADEs, other adverse events (AEs) such as skin injuries (Broom et al., 2019) unplanned extubation, healthcare-associated infections (HAIs), and extravasations (Mccullen and Pieper, 2006) frequently occur in NICUs, often resulting in outcomes distinct from those observed in adults (Dillner et al., 2023). These AEs can prolong hospitalization, increase healthcare costs, and negatively impact neonatal outcomes. The variability in AE incidence is largely attributed to differences in detection methods, inconsistent terminology, and the exclusion of mild or self-limiting events in certain studies (Naessens et al., 2009; Meyer-Massetti et al., 2011). Prematurity and low birth weight (Canto-Rodríguez et al., 2023) are recognized as significant risk factors for AEs, particularly HAIs (Moura et al., 2020; Magluta, 2021), which are among the leading contributors to neonatal morbidity and mortality (Dias et al., 2022). These conditions, coupled with prolonged mechanical ventilation, parenteral nutrition, and invasive procedures, further compound the risks of AEs (Moura et al., 2020; Dias et al., 2022).

NICUs play a pivotal role in addressing these challenges, providing high-complexity care delivered by multidisciplinary teams with specialized training (Batista et al., 2021). However, the incidence, severity, and causes of AEs in this setting remain underexplored, particularly regarding medication safety. Several tools for the identification of AEs have been proposed, each presenting a variety of methods with distinct advantages and limitations. To enhance the efficiency of analyses and ensure more accurate identification, trigger-based approaches have been developed as indicators or warning signals that, when detected, require additional evaluation (Classen et al., 1991; Griffin and Classen, 2008). This approach enables targeted investigation, contributing to the detection of events that might otherwise go unnoticed in traditional analyses (Resar et al., 2006; Sharek et al., 2006) and employed predefined indicators, including abnormal laboratory findings, medication modifications, and clinical signs indicative of potential harm. Medical records flagged by these triggers underwent systematic and detailed analysis to confirm the occurrence of true AEs. However, despite these advancements, the identification of AEs in NICUs remains a complex and underexplored area, particularly concerning medication safety. Addressing this knowledge gap requires rigorous research to monitor drug use in neonatology, identify the causes and consequences of AEs and implement targeted interventions to enhance patient safety.

This study aimed to provide information on the following: 1) Estimate the incidence of neonates in NICUs experiencing AEs using specific trigger tools; 2) Analyze the relationship between the occurrence of AEs and neonatal clinical variables; 3) Estimate the frequency of ADRs and characterize them in terms of causality; 4) Identify drug classes associated with ADRs in NICUs; 5) Evaluate the performance of selected triggers for identifying AEs in neonates in NICUs.

## 2 Methods

### 2.1 Study design, setting, and population

We conducted a retrospective observational study between January 1 and April 30, and between September 1 and 31 October 2021, utilizing data from neonates admitted to the NICU at the Hospital Estadual Sumaré - Dr. Leandro Franceschini, Sumaré, SP, Brazil. This public university hospital serves as a referral center for high-risk pregnancies and deliveries across six regional cities, performing an average of 2,200 deliveries annually. The study population included all neonates admitted to the NICU at Hospital Estadual Sumaré - Dr. Leandro Franceschini during the study periods, regardless of birth weight, gestational age, sex, or diagnostic hypothesis, provided they met the predefined inclusion criteria.

### 2.2 Inclusion and exclusion criteria

Inclusion criteria required that neonates were admitted to the NICU during the period study, had been prescribed at least one medication and had a hospitalization period exceeding 48 h. The neonatal period was defined as the first 28 days of life (World Health Organization, 2014). Exclusion criteria encompassed medical records with incomplete data.

### 2.3 Ethics committee approval

The study was approved by the Ethics Committee of the Universidade Estadual de Campinas, Campinas, SP, Brazil under protocol number 39936920.0.0000.5404. It was conducted in accordance with the ethical principles outlined in the 2013 Declaration of Helsinki for medical research involving human subjects and Resolution No. 510 of 7 April 2016, issued by the Ministry of Health, concerning ethics in human subject research.

### 2.4 Sampling

Following the methodology established by the Institute for Healthcare Improvement (IHI) (Institute for Healthcare Improvement IHI, 2009), we randomly selected 20 medical records per month, resulting in a total of 120 neonates. This sampling strategy is widely recognized in scientific literature as a robust and practical approach for detecting AEs without compromising analytical rigor or overburdening the review process. This sample size has been validated in several studies as sufficient to identify patterns and trends in the occurrence of AEs, allowing for consistent inferences regarding the quality and safety of care without requiring an exhaustive review of all available records. Furthermore, this strategy enables monitoring of temporal variations in AE incidence, which is essential for evaluating the impact of interventions and continuous improvement policies (Institute for Healthcare Improvement IHI, 2009; Child Health Corporation of America, 2007).

The statistical power of this study was calculated using G*Power software (version 3.1.9.4), considering an effect size of 0.30—classified as a medium effect according to Cohen’s conventions (small = 0.10; large = 0.40) and a significance level of 0.05. With a sample of 120 participants and comparative analyses involving a maximum of three groups (sex, gestational age, birth weight, and length of hospital stay), the estimated statistical power was 0.8358, which is considered sufficient for robust statistical inference.

### 2.5 Data collection

Demographic and clinical variables were collected from records. Neonate sex was the demographic variable included. General clinical data included gestational age (≤195 days [extremely preterm]; 196–237 days [moderately preterm]; 238–258 days [late preterm]; 259–293 days [full-term]), birth weight (≤999 g [extremely low birth weight–ELBW]; 1,000–1,499 g [very low birth weight–VLBW]; 1,500–2,499 g [low birth weight–LBW]; 2,500–2,999 g [insufficient weight–IW]; ≥3,000 g [adequate weight–AW]), and length of hospitalization (days).

### 2.6 Record review and method of assessment

A trigger-based methodology was employed, using specific indicators in medical records to signal potential AEs. We applied 17 neonatology-specific triggers derived from previous studies (Sharek et al., 2006; Ventura et al., 2012; Fabretti et al., 2018; Feng et al., 2022), as well as general pediatric trigger frameworks (Takata et al., 2008; Matlow et al., 2011; Unbeck et al., 2014). The final trigger list was developed and refined in consultation with specialists and clinical pharmacists (Sharek et al., 2006; Ventura et al., 2012; Fabretti et al., 2018; Feng et al., 2022; Brasil. Ministério da Saúde. Atenção à Saúde do Recém-Nascido, 2014; Brasil. Agência Nacional de Vigilância Sanitária, 2017; Marba and MezzaCappa, 2008; Martin et al., 2017; Krebs et al., 2020). These indicators include signs, symptoms, abnormal laboratory findings, medication prescriptions and procedure-related complications (Supplementary Table S1).

Two senior reviewers (clinical pharmacists) independently analyzed the medical records using the previously cited trigger-based methodology. In cases of disagreement, a third reviewer was consulted. The data collection followed a randomized sequence, and analysis adhered to the chronological order of events from admission to discharge, transfer, death, or the 28th day of life.

Medical records were accessed through the institutional electronic system. All documents related to each day of hospitalization were reviewed, including admission records, nursing and medical progress notes, prescriptions, and laboratory results. Discharge summaries provided additional information on diagnostic hypotheses, discharge date, death, transfers, surgeries, and procedures.

Medical progress notes and prescriptions were analyzed alongside laboratory results, with particular attention to clinical changes and medication dosage adjustments. Clinical progression, diagnostic hypotheses, and altered laboratory results were analyzed in the context of patient-specific characteristics such as prematurity, physiological adaptation, natural disease progression, and treatment complications. Prescribed medications were evaluated for dosage per kilogram per day and per dose, considering the daily weight of the neonates. Additionally, medication dilutions were verified in accordance with recommendations from the scientific literature.

After trigger identification, medical records were thoroughly reviewed to assess whether the triggers were associated with suspected AEs, ensuring a systematic and evidence-based evaluation. Repeated triggers in the same patient on different dates were counted only once, except for HAIs and antimicrobial use.

### 2.7 Definition and classification of AEs

AEs were defined as incidents arising from healthcare processes that resulted in patient harm (World Health Organization - WHO, 2009). Once an AE was confirmed, additional information was recorded, including the date of first occurrence and severity classification. Two validated severity grading systems were employed: the World Health Organization (WHO) classification (Uppsala Monitoring Centre and Organização Mundial da Saúde, 2005), which categorizes AEs into four levels—mild, moderate, severe, and death—and the Neonatal Adverse Event Severity Scale (NAESS), a tool specifically adapted to neonatal populations (Salaets et al., 2019). The NAESS ranks severity on a five-point scale: grade 1 (mild), grade 2 (moderate), grade 3 (severe), grade 4 (life-threatening), and grade 5 (death) (Salaets et al., 2019). In this study, AEs were further subdivided into non-medication-related and medication-related AEs; however, within the latter category, only ADRs were considered. Other medication-related AEs (e.g., DDIs, medication errors) were not included. ADRs are unintentional and harmful events that occur during the use of standard therapeutic doses of medications for prophylaxis, diagnosis, treatment, or modification of physiological functions (World Health Organization - WHO, 2009).

ADRs were classified according to causality, using the neonatology-specific algorithm (Du et al., 2013), which considers the clinical context and documented evidence in neonates. The likelihood of an association between the medication and the ADR was categorized into four levels based on the final score: definite (≥14), probable (Kaguelidou et al., 2016; Broom et al., 2019; Mccullen and Pieper, 2006; Dillner et al., 2023; Naessens et al., 2009; Meyer-Massetti et al., 2011; Canto-Rodríguez et al., 2023), possible (Lim and Pettit, 2019; Samiee-Zafarghandy et al., 2023; Domingues et al., 2023; De Las Salas and Díaz-Agudelo, 2017), and unlikely (≤2). Additionally, causality was assessed using the WHO criteria, which includes the following categories: certain, probable, possible, unlikely, conditional, and unclassifiable (Uppsala Monitoring Centre and Organização Mundial da Saúde, 2005). Medications associated with ADRs were classified using the Anatomical Therapeutic Chemical (ATC) system (World Health Organization Collaborating Centre for Drug Statistics Methodology, 2024).

### 2.8 Outcome measures

The incidence of patients with AEs was calculated as the proportion of individuals who experienced at least one AE, divided by the total number of patients included in the study, and expressed as a percentage. The rate of AEs was calculated as the number of AEs identified during the study period divided by the total number of patient-days, multiplied by 1,000. Results are expressed as AEs per 1,000 patient-days. Patient-days were calculated by summing the total length of hospital stay (in days) for all patients included in the study.

Trigger frequency was calculated as the number of times a trigger was identified divided by the total number of medical records reviewed, multiplied by 100. The AE occurrence rate was calculated by dividing the number of AEs identified through triggers by the total number of medical records reviewed, also multiplied by 100. The positive predictive value (PPV) of each trigger, expressed as a percentage, represents its ability to detect true AEs. The PPV was determined using the method proposed by Handler et al., which accounts for both the frequency of triggers and the incidence rate of AEs (Handler et al., 2007). It was calculated by dividing the AE occurrence rate by the trigger frequency and multiplying the result by 100, reflecting each trigger’s performance in identifying AEs.

### 2.9 Data analysis

The independent variables were described using absolute and relative frequencies, mean, standard deviation. Triggers and AEs were also reported as absolute and relative frequencies. The occurrence of AEs was expressed for each birth weight in absolute frequencies. To assess the association between birth weight categories and the occurrence of specific AEs, the Chi-square test or Fisher’s exact test was applied, depending on the expected frequencies in each cell counts.

A significant level of 5% was adopted for all analysis. Statistical analyses were performed using R software, version 4.4.2.

## 3 Results

### 3.1 Study population

**FIGURE 1:**
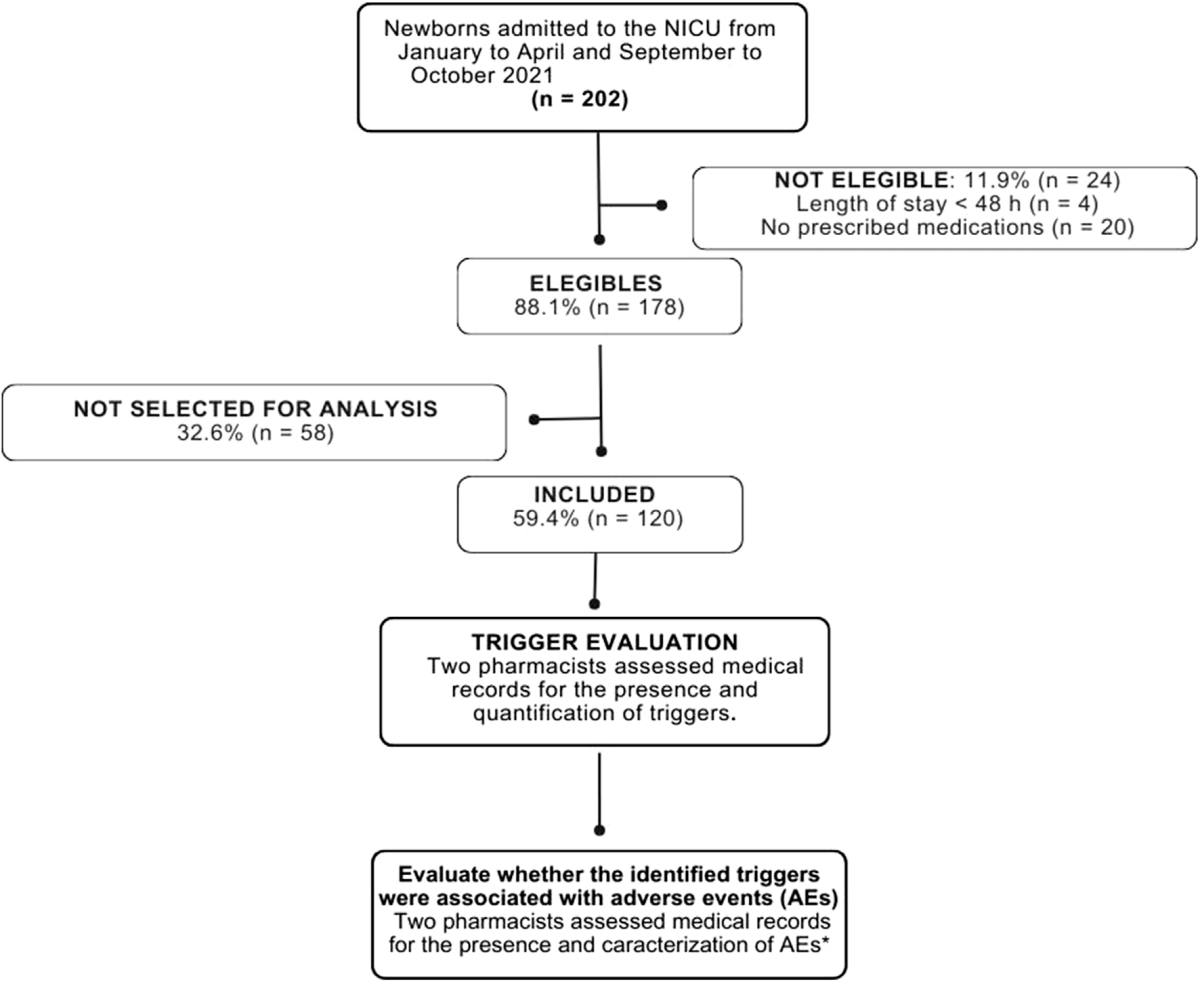
Flow diagram of neonate selection and randomization. NICU: Neonatal Intensive Care Unit. *AEs were defined as incidents arising from healthcare processes that resulted in patient harm (World Health Organization - WHO, 2009). In this study, AEs were further subdivided into non-medication-related and medication-related AEs; however, within the latter category, only ADRs were considered. Other medication-related AEs (e.g., drug interactions, medication errors) were not included. ADRs are unintentional and harmful events that occur during the use of standard therapeutic doses of medications for prophylaxis, diagnosis, treatment, or modification of physiological functions (World Health Organization - WHO, 2009).

During the study period, 202 neonates were admitted to the NICU. After applying inclusion criteria, cases were excluded, leaving 178 eligible for randomization. Each month, 20 neonates were randomly selected for detailed analysis, resulting in a sample of 120 neonates, representing 67.4% of the eligible population (Figure 1).

There was a predominance of male neonates (56.7%). The mean gestational age was 33.6 ± 3.7 weeks, with 76.7% of neonates classified as preterm, including 10.0% with extreme prematurity. Based on birth weight, 77.5% of the participants weighed less than 2,500 g. In terms of length of stay, 50.0% of hospitalizations exceeded 21 days (Table 1). The total number of patient-days was 3,423.

**TABLE 1:** General clinical characteristics of the study population (n = 120).

Variables	% (n)	Mean ± SD
Gestational age (days)		
≤195 days (Extremely Preterm)	10.0 (12)	183.7 ± 9.3
196–237 days (Moderately Preterm	35.0 (42)	222.2 ± 11.0
238–258 days (Late Preterm)	31.7 (38)	245.3 ± 5.7
259–293 days (Full-Term)	23.3 (28)	269.2 ± 7.4
TOTAL	**100.0 (120)**	**235.2** **±** **26.1**
BIRTH WEIGHT 9 g)		
≤999 g (ELBW)	9.1 (11)	779.5 ± 146.3
1,000–1,499 g (VLBW)	16.7 (20)	1301.2 ± 136.5
1,500–2,499 g (LBW)	51.7 (62)	2010.7 ± 265.9
2,500–2,999 g (IW)	10.0 (12)	2708.2 ± 138.3
≥3,000 g (AW)	12.5 (15)	3373.0 ± 330.5
TOTAL	**100.0 (120)**	**2019.6** **±** **750.5**
LENGTH OF STAY (days)		
2–5	5.8 (7)	3.6 ± 1.0
6–10	12.5 (15)	7.8 ± 1.4
11–15	17.5 (21)	13.0 ± 1.2
16–20	14.2 (17)	17.7 ± 1.4
21–27	17.5 (21)	23.2 ± 1.7
≥28	32.5 (39)	57.0 ± 26.0
TOTAL	**100.0 (120)**	**28.5** **±** **25.3**

g, grams; AW, adequate weight; ELBW, extremely low birth weight; IW, insufficient weight; LBW, low birth weight; VLBW, very low birth weight.

### 3.2 Triggers performance

The study identified a total of 249 triggers in 70.8% (n = 85) of the medical records, with a mean of 2.1 ± 2.2 triggers per patient. The number of triggers identified per neonate varied from 0 to 9. The five most frequently identified triggers were hyperglycemia, increased frequency of bowel movements, HAIs, antimicrobial use, and hypotension (Table 2). Flumazenil, hypernatremia, and naloxone were not identified in the reviewed medical records, suggesting either their absence or potential underreporting in clinical documentation (Table 2).

**TABLE 2:** Performance of neonatal adverse event triggers based on Positive Predictive Value.

Triggers	Trigger/100 records	AE/100 records	PPV (%)
Necrotizing Enterocolitis	2.5	2.5	100.0
Accidental Extubation	5.0	5.0	100.0
Hypocalcemia	4.2	4.2	100.0
HAIs	30.8	30.8	100.0
Antimicrobials Use	30.8	30.8	100.0
Hyponatremia	13.3	10.8	81.3
Increased Serum Creatinine	5.0	3.3	66.7
Hypokalemia	7.5	5.0	66.7
Hyperglycemia	38.3	22.5	58.7
Increased Frequency of Bowel Movements	34.2	16.7	48.8
Hyperkalemia	5.8	2.5	42.9
Hypotension	14.2	5.0	35.3
Hypercalcemia	10.0	0.8	8.3
Phenobarbital	5.8	0.0	0.0
Flumazenil	0.0	0.0	0.0
Hypernatremia	0.0	0.0	0.0
Naloxone	0.0	0.0	0.0
TOTAL	**207.5**	**140.0**	**67.5**

HAIs, Healthcare-associated infections; AE, adverse event; PPV, preditive positive value.

The most frequently observed AEs were identified through the triggers HAIs, antimicrobial use, hyperglycemia, increased bowel movement frequency, and hyponatremia (Table 2).

The triggers with the highest performance, based on their PPV, were necrotizing enterocolitis, accidental extubation, hypocalcemia, HAIs, and antimicrobials use, all of which demonstrated a PPV of 100% (Table 2). The hyponatremia trigger also showed good performance, with a PPV of 81.3%. Additionally, triggers such as increased serum creatinine, hypokalemia, and hyperglycemia demonstrated PPVs between 50.0% and 70.0%, indicating a moderate likelihood of detecting true AEs. In contrast, increased frequency of bowel movements, hyperkalemia, hypotension, hypercalcemia, and phenobarbital had PPVs below 50.0%, reflecting a substantial proportion of false-positive results (Table 2). Overall, 168 of the 249 identified triggers were associated with the occurrence of an AE, resulting in a PPV of 67.5% (Table 2).

### 3.3 AEs incidence and characterization

As previously mentioned, a total of 168 AEs were identified through trigger evaluation, with a mean of 1.4 per patient. Half of the neonates (n = 60; 50.0%) experienced at least one AE, and 40.8% (n = 49) had at least one ADR. The incidence was 49.1 per 1,000 patient-days.

Regarding severity, using the WHO classification, 44.6% were considered moderate, 54.8% severe, and 0.6% fatal. No events were classified as mild, suggesting that AEs in the NICU tend to be significant. Based on the NAESS, AEs were classified as follows: 44.0% as grade 2 (moderate), 51.2% as grade 3 (severe), 4.2% as grade 4 (life-threatening), and 0.6% as grade 5 (death). According to the NAESS, increased bowel movement frequency and hyperglycemia were considered moderate (grade 2), along with some HAIs of cutaneous origin. Severe AEs (grade 3) included increased serum creatinine, hypotension, and electrolyte imbalances such as hypercalcemia, hyperkalemia, hypocalcemia, hypokalemia, and hyponatremia. Life-threatening AEs (grade 4) included necrotizing enterocolitis, accidental extubation, antimicrobial use, and 67.4% of identified HAIs. The only fatal case was attributed to necrotizing enterocolitis. Of the total AEs identified (n = 168), 47.6% were categorized as non-medication-related AEs (n = 80), whereas 52.4% were classified as ADRs (n = 88; 40.8% of the included neonates), with a mean of 0.7 ± 1.1 ADRs per patient.

Among the 60 neonates who experienced AEs, 81.7% (n = 49) presented with an ADRs. Of these, 59.2% were male neonates (p = 0.643). The analysis identified 21 drugs responsible for a total of 88 ADRs. Cardiac therapy drugs, used in the management of cardiovascular disorders, accounted for 34.1% of ADRs. Caffeine, utilized for apnea treatment, contributed 27.3%, whereas systemic antimicrobials accounted for 23.9% of ADRs (Table 3).

**TABLE 3:** Medications related to ADRs (n = 88) according to the Anatomical Therapeutic Chemical Classification.

ATC groups	ATC CODE	% (N)
Multivitamins	A11	2.2 (2)
Multivitamins	A11JB	1.1 (1)
Vitamin A, D, E (combination)	A11JA	1.1 (1)
Blood Substitutes and perfusion solutions	**B05**	**7.9 (7)**
50% Glucose	B05CX	6.8 (6)
Potassium chloride	B05XA01	1.1 (1)
Cardiac therapy	**C01; C03; C07; C09**	**34.1 (30)**
Alprostadil	C01EA01	4.5 (4)
Ibuprofen	C01EB16	3.4 (3)
Dobutamine	C01CA07	2.3 (2)
Dopamine	C01CA04	4.5 (4)
Furosemide	C03CA01	12.5 (11)
Propranolol	C07AA05	2.3 (2)
Captopril	C09AA01	4.5 (4)
Systemic antimicrobials	**J01**	**23.9 (21)**
Amikacin	J01GB06	10.2 (9)
Ampicilin	J01CA01	4.5 (4)
Ampicilin + Sulbactam	J01CR01	1.1 (1)
Cefepime	J01DE01	1.1 (1)
Cefalexin	J01DB01	1,1 (1)
Oxacilin	J01CF04	2.3 (2)
Potassium Penicillin G	J01CE01	1.1 (1)
Vancomycin	J01XA01	2.3 (2)
Psychoanaleptics	**N06**	**27.3 (24)**
Caffeine	N06BC01	27.3 (24)
Drugs used in obstructive respiratory diseases	**R03**	**4.5 (4)**
Aminophylline	R03DA06	4.5 (4)

ATC, anatomical therapeutic chemical.

Bold values indicate therapeutic groups according to the second level of the ATC classification and their corresponding absolute and relative frequencies.

The results for the causality assessment using the Du et al. algorithm indicated that 25.1% were probable, 26.4% possible, and 48.5% unlikely. According to the WHO criteria, 7.2% were classified as probable, 44.3% as possible, and 48.5% as unlikely. No ADRs were classified as definitive.

### 3.4 Clinical factors associated with AEs

Of the 60 patients who experienced AE, 55% (n = 33) were male (p = 0.712). The distribution of the number of AEs according to birth weight revealed that 28.0% (n = 47) occurred in neonates with ELBW, 25.0% (n = 42) in those with VLBW, 24.4% (n = 41) in those with LBW, 15.5% (n = 26) in infants with IW, and 7.1% (n = 12) in those with AW (p < 0.0001). Table 4 details the distribution of positive triggers stratified by birth weight category.

**TABLE 4:** Distribution (%) of adverse events identified by triggers according birth weight.

Positive triggers	<999 g ELBW	1,000–1,499 g VLBW	1,500–2,499 g LBW	2,500–2,999 g IW	≥3,000 g AW	p-value^a^
Increased Serum Creatinine	75.0	0.0	0.0	25.0	0.0	0.07
Increased Frequency of Bowel Movements	10.0	30.0	35.0	15.0	10.0	0.24
Necrotizing Enterocolitis	66.7	0.0	0.0	33.3	0.0	0.25
Accidental Extubation	50.0	16.7	16.7	16.7	0.0	0.41
Hypercalcemia	100.0	0.0	0.0	0.0	0.0	
Hyperkalemia	0.0	33.3	33.3	33.3	0.0	0.40
Hyperglycemia	22.2	33.3	33.3	7.4	3.8	0.03
Hypocalcemia	0.0	40.0	60.0	0.0	0.0	0.09
Hypokalemia	33.3	33.3	33.3	0.0	0.0	0.41
Hyponatremia	46.2	38.5	0.0	7.7	7.7	0.02
Hypotension	66.7	0.0	33.3	0.0	0.0	0.03
HAIs	24.3	21.6	21.6	21.6	10.8	0.73
Antimicrobial use	24.3	21.6	21.6	21.6	10.8	0.73
TOTAL	28.0	25.0	24.4	15.5	7.1	

^a^ Chi-square test.

AW, adequate weight; ELWB, extremely low birth weight; g, grams; HAIs, Healthcare-associated infections; IW, insufficient weight; LBW, low birth weight; VLBW, very low birth weight.

According to the NAESS, grade 3 (severe) AEs were most frequent among ELBW (70.2%) and VLBW (54.8%) neonates. Conversely, neonates with IW and AW exhibited higher proportions of grade 2 (moderate) AEs (50.0% and 58.3%, respectively). Grade 4 (life-threatening) AEs were predominantly observed in ELBW (8.5%) and IW neonates (3.8%). One fatal case (grade 5) was reported in the IW group (3.8%) (p = 0.015).

The incidence of neonates experiencing at least one AE varied according to gestational age, with rates of 75.0% for extremely preterm, 54.8% for very preterm, 44.7% for late preterm, and 39.3% for term neonates (p = 0.036). Overall, 82.7% (n = 139) of AEs were identified in neonates with some degree of prematurity, with 46 occurring in extremely preterm neonates, 61 in very preterm, 32 in late preterm, and 29 in term neonates (p = 0.012). Among extremely preterm neonates, the most common complications included increased serum creatinine (75.0%), accidental extubation (66.7%), hypercalcemia (100.0%), hyponatremia (53.8%), and hypotension (50.0%).

Regarding the length of hospital stay, the incidence rates for the 2–5 days and 11–15 days intervals were identical, at 14.3%. Similar rates were observed for stays of 6–10 days and 16–20 days (46.7% and 47.1%, respectively). The highest incidence was found in hospitalizations longer than 28 days (76.9%), followed by the 21–27 days interval, during which 52.4% of patients experienced at least one AE (p < 0.001).

## 4 Discussion

In this study, a predominance of male neonates was observed among those admitted to the NICU, with a mean gestational age of 33.6 weeks. The majority of neonates were preterm (76.7%), with 10% classified as extremely preterm. This clinical profile aligns with findings in Brazil that also report a higher frequency of prematurity and a predominance of male sex among NICU admissions international research has emphasized the increased susceptibility of preterm neonates to critical conditions requiring intensive care (Ventura et al., 2012; Ligi et al., 2008).

The observed average length of hospital stay among the neonates in this study was 28.5 days, a duration that is consistent with findings reported in international literature, although variability exists depending on gestational age, birth weight, and the complexity of clinical conditions. Similarly, research by Oza et al. (2013) encompassing data from low- and middle-income countries, highlighted prolonged hospital stays—often exceeding four weeks—among neonates with VLBW or severe complications, emphasizing the burden on healthcare systems in resource-limited settings. Moreover, longer hospital stays have been critically associated with increased risk of adverse outcomes, including hospital-acquired infections (Stoll et al., 2010) reinforcing the importance of implementing targeted strategies to reduce length of stay without compromising safety (Ravi et al., 2019).

A high variability was observed in the number of triggers identified, with a mean of 2.2 ± 2.4, a finding consistent with previous studies (Sharek et al., 2006; Ventura et al., 2012; Feng et al., 2022). In contrast, Fabretti et al. (2018) reported a significantly higher average of 7.4 triggers per patient, using an expanded set of 48 triggers. This discrepancy in identification frequency may be attributed to differences in the trigger sets applied (Pierdevara et al., 2017), which range from general AEs (Dillner et al., 2023; Sharek et al., 2006; Ventura et al., 2012), ADEs (Fabretti et al., 2018), non-specific categories (Dillner et al., 2023), medication errors (Maziero et al., 2021), and specific clinical conditions such as nasal injuries and thermoregulation disorders (Ventura et al., 2012).

The performing triggers in detecting AEs were evaluated using both overall and individual PPVs. Triggers with low PPVs or that identify events less frequently may decrease the overall performance of the method (Classen et al., 1991; Giordani et al., 2012). The overall PPV was 67.5%, consistent with the findings of Feng et al., (2022) and higher than those reported in other studies, which ranged from 22.5% to 38.0% (Sharek et al., 2006; Fabretti et al., 2018; Unbeck et al., 2014).

These variations may be explained by differences in population characteristics, such as gestational age and birth weight, as well as the specificity and composition of the trigger sets used. High-performing triggers—such as necrotizing enterocolitis, accidental extubation, hypocalcemia, HAIs, and antimicrobial use—achieved a PPV of 100.0%. This may be due to the fact that these represent direct AEs and are commonly used as quality-of-care indicators (Sharek et al., 2006; Snijders et al., 2009). In specific cases of hypocalcemia, high PPV may be associated with the off-label use of furosemide in preterm neonates for the treatment of symptomatic patent ductus arteriosus (Backes et al., 2022; Vuralli, 2019). Necrotizing enterocolitis showed a higher PPV than those reported in other investigations possibly due to differences in diagnostic classification criteria and the clinical characteristics of the neonatal subgroups studied (Sharek et al., 2006; Fabretti et al., 2018).

Hyponatremia also stood out, with a PPV of 81.3%, higher than that reported by Fabretti et al. (2018). Previous studies analyzed electrolyte abnormalities as grouped conditions rather than as specific triggers, which may reduce accuracy in detecting individual events (Sharek et al., 2006; Barrionuevo and Esandi, 2010).

Among intermediate-performing triggers, elevated serum creatinine and hypokalemia both had a PPV of 66.7%. Variability in PPVs for creatinine across studies (11.0%–100.0%) likely reflects differences in diagnostic criteria, monitoring frequency, and nephrotoxic drug exposure (Sharek et al., 2006; Fabretti et al., 2018). Hyperglycemia, an established risk factor for neonatal mortality (Mesotten et al., 2018), showed a PPV of 58.7%, similar to Fabretti et al. (2018) but lower than Sharek et al. (2006). Its detection may have been limited by demand-based rather than continuous monitoring and the absence of a standardized definition in neonates.

Variations in definitions and thresholds for electrolyte disturbances also affected trigger performance. For example, hyperkalemia was inconsistently defined, with serum potassium cutoffs ranging from >5.5 to >8.0 mEq/L across studies (Sharek et al., 2006; Li et al., 2014; Ni et al., 2018), hindering comparability.

Triggers with PPVs below 50%—such as increased frequency of bowel movements, hyperkalemia, hypercalcemia, hypotension, and phenobarbital—often reflect clinical variability rather than true AEs. Diarrhea had limited utility due to inconsistent bowel patterns and reliance on documentation. Hypotension showed low predictive value (35.2%), likely influenced by infrequent recordings and lack of gestational age-adjusted criteria. The absence or low yield of triggers such as flumazenil, naloxone, and hypernatremia may result from appropriate medication use, incomplete records, or the limited sample size.

The trigger set identified 168 AEs, averaging 1.4 events per patient, aligning with prior findings (1.7 events) (Feng et al., 2022), but exceeding rates from studies with narrower trigger sets or different populations (Sharek et al., 2006; Fabretti et al., 2018). The AE rate of 49.1 per 1,000 patient-days was also higher than previously reported (Sharek et al., 2006; Yalçın et al., 2022), which may reflect differences in definitions, sample characteristics, inclusion of specific triggers (e.g., electrolyte disturbances), and documentation practices.

Approximately 50.0% of neonates experienced at least one AE, a rate comparable to other studies (Fabretti et al., 2018; Feng et al., 2022). The scope of the trigger set directly influences the incidence observed, as demonstrated by Sharek et al. (2006), who reported a 74.0% incidence when including death and cardiopulmonary arrest, and by Cossul et al. (2021), who found a 70.0% incidence by considering technical complaints, medication errors, and injuries related to central and peripheral venous access. Ventura et al. (2012), in a prospective analysis reported a high rate of 84.0%.

The frequency of AEs like HAIs observed in this study was comparable to findings from other investigations using similar definitions (World Health Organization, 2014; Institute for Healthcare Improvement IHI, 2009; Child Health Corporation of America, 2007), although variations were noted due to differences in diagnostic criteria and identification methods. Hyperglycemia had a prevalence of 16.1%, with substantial variability across studies (Ventura et al., 2012). The incidence of increased frequency of bowel movements was lower than that reported in other studies (Fabretti et al., 2018; Feng et al., 2022), possibly reflecting differences in standardization criteria and assessment methods. Accidental extubation may have been underestimated, potentially due to the retrospective nature of the study. Hypotension and elevated serum creatinine levels showed frequencies consistent with the literature but were influenced by varying definitions and monitoring approaches. Necrotizing enterocolitis was infrequent, likely due to dependence on clinical diagnosis and proper documentation. No adverse events were identified related to the use of phenobarbital, naloxone, or flumazenil, despite their inclusion as triggers in previous studies.

ADR frequency reached 40.8%, with a mean of 0.7 per patient, consistent with larger neonatal cohorts (Kaguelidou et al., 2016). Cardiovascular drugs—especially furosemide, dopamine, and captopril—were the main culprits, as previously reported (Leopoldino et al., 2023; Sugioka et al., 2020; Workineh and Workie, 2022). Caffeine, widely used for apnea of prematurity, accounted for nearly a third of ADRs, while antibiotics were linked to most cases of diarrhea and electrolyte imbalances, while ibuprofen was linked to a case of necrotizing enterocolitis. Causality analysis using the algorithm by Du et al. showed lower ADR rates in all categories compared to earlier studies, except for the “unlikely” category. No ADRs were classified as definite, which may be related to the absence of documented practices such as drug detection in blood or fluids and the use of doses above recommended levels (Du et al., 2013; Sugioka et al., 2020).

Lower gestational age and reduced birth weight are well-established risk factors for the occurrence of AEs in the neonatal population, as the physiological immaturity characteristic of these subgroups predisposes them to greater susceptibility to clinical complications and drug-induced toxicity (Canto-Rodríguez et al., 2023; Giesinger and McNamara, 2016).

In the present study, a higher prevalence of AEs was observed among extremely preterm and very preterm neonates, as well as those classified as having ELBW and VLBW, corroborating existing literature that links these factors to increased clinical vulnerability (Srulovici et al., 2012). Among the AEs evaluated, hyperglycemia, hyponatremia, and hypotension demonstrated statistically significant associations when stratified by birth weight, reinforcing the relationship between low birth weight and a heightened predisposition to metabolic and hemodynamic disturbances (Inage et al., 2022). Hyperglycemia was notably more prevalent in neonates weighing less than 2,500 g. This finding aligns with previous studies that associate prematurity and intrauterine growth restriction with altered glucose homeostasis, potentially driven by immature insulin secretion, exaggerated stress responses, and the frequent use of parenteral nutrition in this population (Inage et al., 2022; Beardsall, 2021).

Hyponatremia was similarly more frequent among ELBW and VLBW neonates, likely reflecting the interplay of renal immaturity, altered fluid homeostasis, and pharmacological interventions such as diuretic administration (Hao, 2019). These data highlight the importance of vigilant electrolyte monitoring in high-risk neonates to mitigate further clinical deterioration. Hypotension, significantly more prevalent in ELBW infants, underscores the inherent hemodynamic instability of this subgroup, necessitating individualized cardiovascular monitoring and support strategies. Moreover, a prolonged duration of hospitalization was associated with an increased prevalence of AEs, particularly in neonates with NICU stays exceeding 28 days. Extended hospitalization inherently increases exposure to invasive procedures, broad-spectrum antimicrobials, and nosocomial pathogens, thereby amplifying the cumulative risk of AEs. These findings emphasize the critical need for preventive measures and tailored surveillance protocols for neonates with prolonged NICU admissions (Bartelink et al., 2006).

This study is subject to several methodological limitations that warrant consideration. The retrospective design, coupled with exclusive reliance on data extracted from electronic medical records, inherently constrains the detection of AEs that were either undocumented or insufficiently recorded. As such, the findings are intrinsically dependent on the accuracy, completeness, and consistency of clinical documentation by healthcare professionals, introducing potential information bias. Moreover, the study sample was derived from a single center and may not be representative of other neonatal care settings or broader pediatric populations, thereby limiting the external validity and generalizability of the results. The temporal scope of data collection also constitutes a relevant constraint, as the selected study period may not capture seasonal trends or temporal shifts in clinical practices and institutional protocols. Additionally, the review of medical records was not conducted across consecutive months due to adjustments in the research protocol necessitated by restrictions related to the COVID-19 pandemic. This discontinuity may have introduced variability in case selection and hindered a more homogeneous temporal analysis of AE occurrence.

The findings of this study highlight the complexity and fragility of neonatal care in intensive care settings, particularly among highly vulnerable populations such as preterm and ELBW neonates. The high frequency of AEs and ADRs, combined with variability in the performance of the trigger tools used, underscores not only the sensitivity of the methodology but also the inherent challenges in accurately identifying such events in real-world clinical contexts.

The high PPV observed for certain triggers—such as accidental extubation, necrotizing enterocolitis, and HAIs—demonstrates their potential as robust indicators of neonatal care quality. However, the heterogeneity in the performance of intermediate and low-yield triggers reveals the need for ongoing refinement of trigger sets to enhance their specificity and clinical utility, particularly in neonatal subpopulations at higher risk.

Furthermore, the observed association between physiological immaturity, prolonged hospitalization, and increased prevalence of AEs reinforces the importance of individualized clinical strategies guided by evidence-based practices. Early interventions, rigorous monitoring of hemodynamic and metabolic parameters, and judicious pharmacological management are essential to mitigate risks and promote patient safety.

## 5 Conclusion

In summary, this study offers important contributions to the understanding of neonatal patient safety in intensive care environments, demonstrating the feasibility of using trigger-based tools for the systematic detection of AEs and ADRs. The results suggest that the adoption of standardized approaches, adapted to the specific context of neonatal units, can strengthen surveillance systems and foster sustained improvements in care quality. Nevertheless, further research, particularly through prospective and multicenter designs, is essential to validate these findings and broaden them generalizability. Ultimately, the integration of clinical best practices, structured monitoring tools, and an institutional culture focused on neonatal patient safety represents a promising path toward reducing preventable harm and ensuring safer, more effective, and humanized care.

## Data Availability

The original contributions presented in the study are included in the article/Supplementary Material, further inquiries can be directed to the corresponding author.

## Nomenclature

AE: Adverse Event
ADE: Adverse Drug Event
ADR: Adverse Drug Reaction
AW: Adequate weight
DDI: Drug Interactions
ELBW: Extremely low birth weight
HAIs: Healthcare-associated infections
IHI: Institute for Healthcare Improvement
IW: Insufficient weight
LBW: Low birth weight
NAESS: Neonatal Adverse Event Severity Scale
NICU: Neonatal Intensive Care Unit
PPV: Positive Predictive Value
VLBW: Very low birth weight
