# Supplementary material for: Adverse events in the neonatal intensive care unit identified by triggers
Source: Front Pharmacol. 2025 May 30;16:1539687. doi: 10.3389/fphar.2025.1539687 (PMC12163318; doi:10.3389/fphar.2025.1539687)
Supplement: Supplementary file 1 [file Table1.pdf]

Supplementary Material

Supplementary Table 1. Definitions of Triggers and Indicators Used in the Study.

TRIGGERS	DEFINITIONS
INCREASE IN SERUM CREATININE	Serum creatinine > 1.0 mg/dL or documentation of renal failure or acute kidney injury in the medical record.
INCREASED FREQUENCY OF BOWEL MOVEMENTS	Abnormal stools or frequency ≥ 6 times per day.
NECROTIZING ENTEROCOLITIS	Neonatal condition is characterized by extensive mucosal ulceration, pseudomembrane formation, submucosal hemorrhage, and intestinal necrosis.
UNPLANNED EXTUBATION OR ACCIDENTAL EXTUBATION	Any unintended removal of the endotracheal tube.
PHENOBARBITAL	Prescription of a loading dose of 10–20 mg/kg/dose.
FLUMAZENIL	Prescription of flumazenil.
HYPERCALCEMIA	Ionized calcium > 5.81 mg/dL or ≥ 1.45 mEq/L, or documentation of hypercalcemia in the medical record.
HYPERKALEMIA	Potassium > 6.0 mEq/L for term neonates or > 6.5 mEq/L for preterm neonates, or documentation of hyperkalemia in the medical record.
HYPERGLYCEMIA	Blood glucose > 125 mg/dL or documentation of hyperglycemia in the medical record.
HYPERNATREMIA	Sodium > 150 mEq/L or documentation of hypernatremia in the medical record.
HYPOCALCEMIA	For preterm neonates < 1500 g: total calcium < 7 mg/dL or ionized calcium < 4 mg/dL or < 1 mEq/L; For term or preterm neonates ≥ 1500 g: total calcium < 8 mg/dL or ionized calcium < 4 mg/dL or < 1.1 mEq/L; or documentation of hypocalcemia.
HYPOKALEMIA	Potassium < 3.0 mEq/L or documentation of hypokalemia in the medical record.
HYPONATREMIA	Sodium < 130 mEq/L or documentation of hyponatremia in the medical record.
HYPOTENSION	Mean Arterial Pressure (MAP):<br>< 30 weeks GA: MAP < 25 mmHg<br>30–35 weeks GA: MAP < 30 mmHg<br>> 35 weeks GA: MAP < 35 mmHg<br>Also consider terms such as hypotensive, or blood pressure drop documented in the medical record.
HAI	Infection with diagnostic evidence (clinical, laboratory, or microbiological) occurring after the first 48 hours of NICU hospitalization.
NALOXONE	Prescription of naloxone.
USE OF ANTIMICROBIALS	Any antibiotic prescribed during hospitalization, including surgical prophylaxis (excluding prophylactic fluconazole), 48 hours after admission.

GA: gestational age; HAI: healthcare-associated infection; MAP: Mean arterial pressure; NICU: neonatal intensive care unit.
